# REEV SENSE IMUs for Gait Analysis in Stroke: A Clinical Study on Lower Limb Kinematics

**DOI:** 10.3390/s25165123

**Published:** 2025-08-18

**Authors:** Thibault Marsan, Sacha Clauzade, Xiang Zhang, Nicolas Grandin, Tatiana Urman, Evan Linton, Ingy Elsayed-Aly, Catherine E. Ricciardi, Robin Temporelli

**Affiliations:** 1 REEV SAS, 31670 Labège, France; sacha.clauzade@gmail.com (S.C.); nicolas.grandin.perea@pm.me (N.G.); ingyhn@gmail.com (I.E.-A.); robin.temporelli@reev.care (R.T.); 2 Center for Clinical and Translational Research, Massachusetts Institute of Technology, Cambridge, MA 02142, USA; xzhang88@mit.edu (X.Z.); tlevkovich@gmail.com (T.U.); linton@mit.edu (E.L.); c_ricci@mit.edu (C.E.R.); 3 Institute for Medical Engineering & Science, Massachusetts Institute of Technology, Cambridge, MA 02142, USA

**Keywords:** gait analysis, inertial sensors, kinematics, stroke, system validation

## Abstract

**Highlights:**

**What are the main findings?**

- REEV SENSE IMUs provide accurate knee kinematics during post-stroke gait.
- The system adapts to various assistive device conditions, with robust knee measurements.

**What are the implications of the main findings?**

- Enable reliable wearable-based gait monitoring outside the laboratory settings.
- Support the broader use of wearable sensors in stroke rehabilitation.

**Abstract:**

Human gait analysis is essential for clinical evaluation and rehabilitation monitoring, particularly in post-stroke individuals, where joint kinematics provide valuable insights into motor recovery. While optical motion capture (OMC) is the gold standard, its high cost and restricted use in laboratory settings limit its accessibility. This study aimed to evaluate the accuracy of REEV SENSE, a novel magnetometer-free inertial measurement unit (IMU), in capturing knee and ankle joint angles during overground walking in post-stroke individuals using assistive devices. Twenty participants with chronic stroke walked along a 10-m walkway with their usual assistive device (cane or walker), while joint kinematics were simultaneously recorded using OMC and IMUs. Agreement between the systems was assessed using the mean absolute error, root mean square error, 95% confidence intervals, and Pearson’s correlation coefficient. Knee angles measured with the IMUs showed a strong correlation with the OMC (r > 0.9) and low errors (MAE < 5°), consistent with clinical acceptability. Ankle angle accuracy was lower for participants using walkers, while knee measurements remained stable regardless of the assistive device. These findings demonstrate that REEV SENSE IMUs provide clinically relevant kinematic data and support their use as a practical wearable tool for gait analysis in real-world or remote clinical settings.

## 1. Introduction

Human Gait Analysis (HGA) plays a crucial role in various domains, including ergonomics, sports performance, and robotics [1,2]. In the clinical field, HGA is particularly significant for assessing the severity of illness in post-stroke survivors [3], providing essential insights that aid in clinical diagnosis and rehabilitation planning [4]. However, the current methods for performing HGA are highly specialized and require substantial expertise, which presents challenges for widespread adoption.

The gold standard for HGA is with an Optical Motion Capture (OMC) system [5]. This configuration enables the accurate computation of lower limb joint kinematics and spatiotemporal parameters, which are essential for clinical assessment [6]. Despite their precision, OMC systems exhibit several notable limitations that restrict their accessibility and practicality. The financial expense of an OMC system is considerable, rendering it inaccessible to most clinics. In addition, the system is bulky and fixed; therefore, it can only be used in laboratories, which limits its usefulness in clinics and research. Furthermore, the system setup and operation require specialized expertise, which is not always readily available, particularly in non-research environments. Finally, the data analysis phase of the OMC system, in contrast to reporting, is time-intensive, further diminishing its efficiency.

To address these limitations, the purpose of this study is to develop and evaluate methodologies for performing automatic HGA outside laboratory conditions. Camera-based systems offer a more accessible alternative to traditional OMC systems; however, they are still impeded by factors such as occlusions, variable lighting conditions, and challenges in estimating joint positions in pathological gait patterns [6].

As an alternative approach, Inertial Motion Capture (IMC) systems have gained attention. These systems use Inertial Measurement Units (IMUs) composed of accelerometers, gyroscopes, and magnetometers. The fusion of data from these sensors, typically through Kalman filters, facilitates the reconstruction of orientation in three-dimensional space [7]. However, the incorporation of magnetometers presents challenges due to their susceptibility to magnetic interference caused by nearby metallic objects, resulting in miscalibration issues. Magnetic disturbances can indeed affect IMUs by distorting magnetometer readings, leading to inaccurate orientation or heading estimates [6].

Recent studies have shown that both IMC-based- and OMC-based systems can be used to assess gait in post-stroke patients. Marker-based systems remain the gold standard for gait analysis in clinical settings due to their higher accuracy in detecting small movement differences in post-stroke walking. In contrast, IMC-based systems offer a portable and cost-effective solution, although they may be less sensitive to complex gait abnormalities [8]. IMC systems are promising for gait analysis in stroke survivors; however, spasticity or hemiparesis can cause misalignment or calibration issues, reducing accuracy compared to marker-based systems [9].

There has been growing interest in custom-built IMUs for clinical gait analysis, with several studies validating new systems against OMC. González-Alonso et al. [10] presented a wireless IMU system designed for real-time 3D orientation tracking with up to ten sensors operating at 50 Hz and communicating over 2.4 GHz, integrating accelerometers, gyroscopes, and magnetometers. He et al. [11] validated a wearable IMU system in healthy female participants and reported excellent test-retest reliability (Intraclass Correlation Coefficient > 0.9) for spatiotemporal gait parameters compared to the OMC system. Similarly, Zhang et al. [12] evaluated a custom IMU system in patients with knee osteoarthritis, reporting RMSE values of 2.6° to 2.7° for the hip and knee angles. Ahmed et al. [13] developed a modular IMU processing pipeline using OpenSim and quaternion-based filtering, demonstrating excellent agreement with the OMC system (Coefficients of multiple correlation > 0.99) in participants with and without transfemoral amputation. Riglet et al. [14] assessed embedded IMU insoles and confirmed their reliability for clinical gait parameter assessment. Al Borno et al. [15] evaluated the accuracy of OpenSense, an open-source IMU-based tool integrated with OpenSim [16], for estimating lower limb joint angles in post-stroke patients, and reported RMSEs below 5.5° for the knee and ankle compared to optical motion capture, demonstrating promising validity in clinical gait analysis. These studies highlight the clinical relevance and growing feasibility of IMU-based gait analysis solutions as portable and cost-effective alternatives to traditional motion capture systems.

Despite their growing use in clinical gait analysis, IMC systems still face key limitations, such as sensitivity to magnetic disturbances, calibration drift, and reduced accuracy in pathological gait analysis. These challenges hinder their clinical applicability, particularly in post-stroke assessments, where precision and repeatability are critical [6]. Next-generation IMU systems aim to address these issues by improving robustness against environmental interference, enhancing sensor fusion algorithms, and optimizing ergonomics for real-world clinical use.

Therefore, REEV SENSE IMUs (REEV SAS, Labège, France), which have been approved by the FDA as a class 1 medical device, were developed to objectively assess patients’ rehabilitation outcomes using motion analysis sensors and to streamline clinical assessment. These motion sensors can be easily and quickly attached to the patient’s shoe, shin, or thigh, enabling the measurement of characteristic biomechanical parameters related to the patient’s motor pattern. They can be used independently by clinicians as they are paired with the REEV SENSE app, which displays clear results.

The REEV SENSE IMUs were paired with the REEV SENSE application to provide an easy-to-use clinical tool for gait analysis. Depending on the sensor placement, the system enables the measurement of either the knee angle when the IMUs are placed on the thigh and shin or the ankle flexion angle when placed on the shin and shoe. This setup allows physical therapists to quickly deploy a ready-to-use solution and obtain accurate gait measurements within a few minutes.

Maintaining accuracy when using assistive devices is crucial because these tools often alter natural gait patterns and can introduce additional sources of variability in the sensor signals. In clinical populations, such as post-stroke individuals, assistive devices like canes, walkers, or orthoses are commonly used, making it essential for wearable sensors to provide reliable data under these conditions. Patients with stroke often present with asymmetric and altered movement patterns, requiring more sensitive tools to detect subtle impairments and track progress. This study specifically focused on stroke survivors to evaluate the robustness of IMU-based gait assessments in a population in which accurate monitoring is key to guiding rehabilitation and clinical decision-making.

Therefore, the objective of this study was to assess the performance of a mobile sensor-based gait analysis system (REEV SENSE) in comparison to an OMC system, with a specific focus on its ability to accurately measure lower limb joint kinematics in post-stroke individuals with different types of assistance devices. In this study, it was hypothesized that the REEV SENSE IMUs could provide accurate estimations of lower limb kinematics during gait compared to optical motion capture systems. Specifically, the differences between the two systems were expected to remain below 5°, which is a commonly accepted threshold in the literature for system validation [17]. We also hypothesized that the accuracy of IMU-derived measurements would be influenced by the use of assistive devices, such as canes or walkers, particularly in post-stroke patients.

## 2. Materials and Methods

### 2.1. Participants

Twenty individuals in the chronic phase of post-stroke recovery (>6 months post-stroke) participated in this study (Table 1). All patients were over 18 years of age and able to walk with or without assistance. Based on the personal assistive devices used during the study, the participants were grouped into three cohorts: eight used a cane (Cane), two used a walker (Walker), and ten walked without any assistive device (None). Each participant consistently used their own device throughout all trials.

During the experiment, each participant was asked to walk at a self-selected speed on a 10-m path and complete up to three round trips. The participants were allowed to use a cane or walker that they used in their daily lives. The protocol lasted approximately an hour for each participant. An average of 25.8 steps per participant was recorded during the experiment (Table 2).

### 2.2. Consent

Recruitment was carried out via Clinical Connection and an online platform, with eligibility determined by the staff at the Center for Clinical and Translational Research (CCTR) at MIT. This study is part of the REEV SENSE project, which focuses on gait analysis in post-stroke impairment (SENS-AG, NCT06234878), and received ethical approval from the Institutional Review Board (IRB Protocol #20234678). Before participating in the informed consent process over the phone or via a video platform, eligible participants received a copy of the consent form via email. Following the consent discussion, participants had sufficient time to ask questions and make informed decisions regarding their participation. Study consent was obtained electronically using REDCap, a HIPAA-compliant research electronic data capture system. A copy of the signed consent form was provided to the participants via email or as a physical copy. Participant identification was confirmed at the end of the consent process and again during the study visit.

### 2.3. Instrumentation

**Figure 1:**
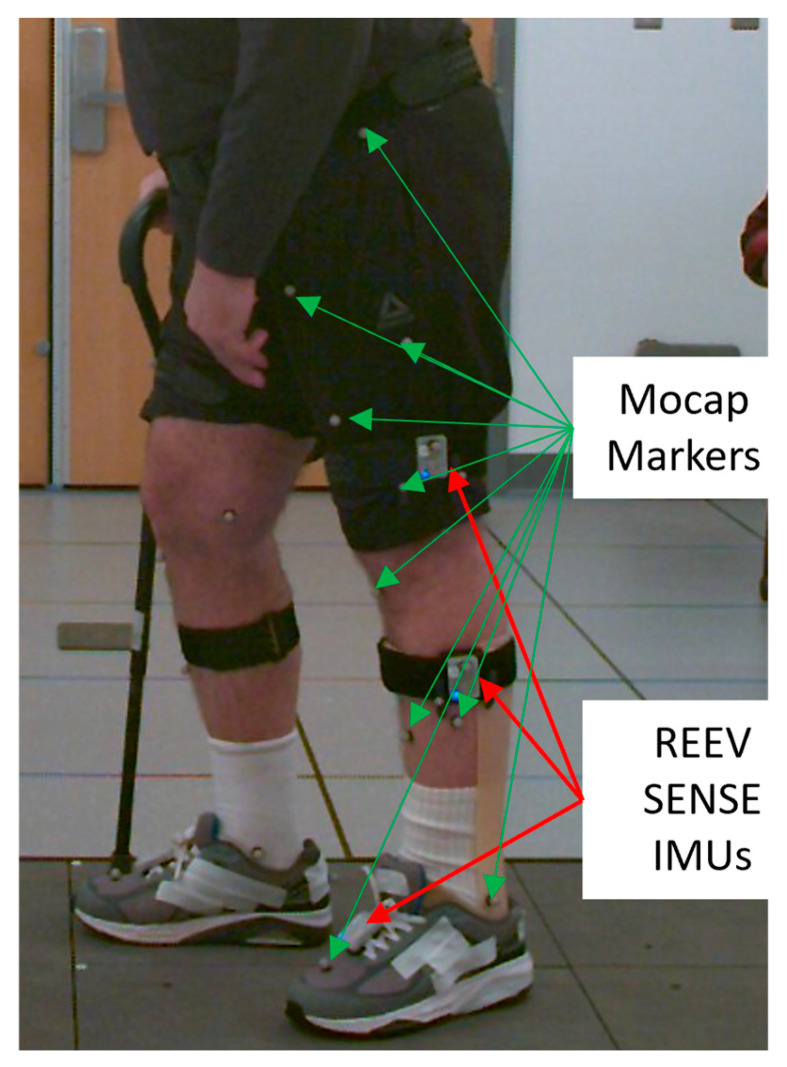
Example of IMU and marker placement on a participant, only one side is seen, and the IMUs were placed symmetrically on the other side.

Kinematic data from the IMC and OMC were recorded simultaneously. For the OMC data, 12 infrared cameras (Qualisys, Göteborg, Sweden) operating at a sampling frequency of 100 Hz captured the participants’ movements using 28 reflective markers (Figure 1) corresponding to the lower body Common Gait Model 2.3 marker set [18]. The IMC system consisted of six REEV SENSE IMUs (REEV SAS, Labège, France), which are based on the LSM6DSL IMU from STMicroelectronics. The 3D linear acceleration was recorded with a range of ±16 g (with g being the acceleration due to gravity g = 9.81 m.s^−2^ here) and a sensitivity of 0.488.10^3^ g at 100 Hz. The 3D angular velocities were recorded using gyrometers at 100 Hz with a range of ±2000°/s and a sensitivity of 0.07°/s. The IMU specifications reported here are based on the manufacturer’s data.

The REEV SENSE IMUs did not include a magnetometer in order to avoid errors caused by magnetic disturbances, which are common in clinical environments and can significantly affect orientation estimates in traditional IMU systems.

Once the participants were equipped with the reflective markers, the REEV SENSE IMUs were placed on the participants with anti-slip straps on the thighs, calves, and shoes (Figure 1). The IMUs placed on the legs were oriented parallel to the sagittal plane and positioned 15 cm from the knee joint. The IMUs on the shoes were attached using magnetic plates placed between the shoelaces. The IMC system communicated with a computer running the REEV SENSE PC application via Bluetooth. To minimize the time required for each participant, all six sensors were placed at once and connected to the REEV SENSE PC application, which enabled simultaneous recording from all sensors.

### 2.4. Data Processing

**Figure 2:**
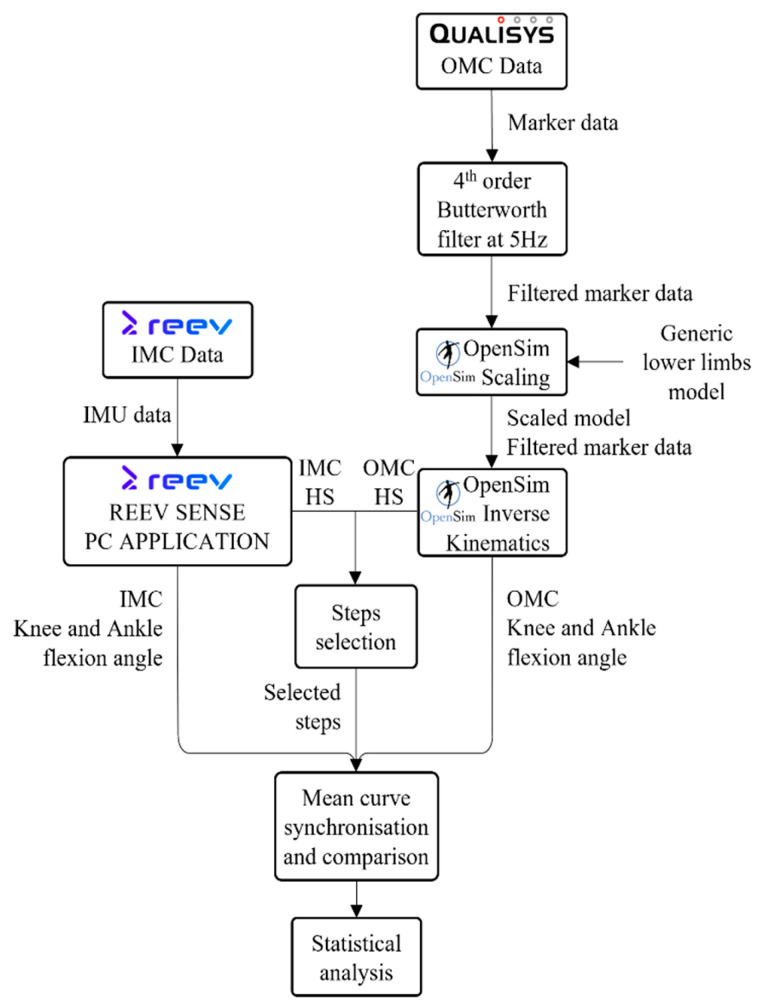
Workflow of data processing. HS: Heel Strike.

OMC and IMC data were collected in parallel and processed separately using OpenSim. Both datasets were used to compute the joint angles. After step selection, the mean curves were synchronized and compared using statistical analyses. The data processing workflow is shown in Figure 2.

The marker data were labeled using the Qualisys Track Manager (version 2023.3). The marker trajectories were then filtered using a 4th-order Butterworth filter set at 5 Hz. OpenSim 4.5 [16] was used to compute the kinematic data. A scaling procedure was first performed to ensure that the model matched the participants’ anthropometry. Subsequently, the Inverse Kinematics tool of OpenSim was used to obtain the knee and ankle angles of the participants during acquisition. Heel strikes and toe-offs were identified from the OMC data using the Relative Distance between Sacral and Foot markers (RDSF) method. This approach was selected based on its demonstrated accuracy compared to the gold standard, force platforms [19].

The IMC data were processed using the REEV SENSE PC application, which provided measurements of knee and ankle flexion. To synchronize the six REEV SENSE IMUs, a custom-built device that performed controlled back-and-forth translational movements was used. All IMUs were initially mounted on this device, and following the synchronization procedure, they were transferred to the appropriate locations on the participants’ lower limbs for data collection. To enable synchronization between the IMC and OMC systems, reflective markers were attached to the custom device and tracked using OMC cameras.

All signals were cropped at the frame corresponding to the end of the first translation of the custom device, which was the beginning of the acquisition. It was also cropped at the beginning of the second translation, which corresponded to the end of acquisition. The REEV SENSE application provided pre-segmented steps between two heel strikes. To ensure that the same steps were selected between the IMC and OMC, the heel strikes of both systems were compared. If a corresponding heel strike existed in both segmentations, with a maximal timing difference of 20 frames, the step was selected for further analysis. This threshold was chosen because it provided a good balance between temporal precision and tolerance to minor detection shifts. Once the steps were selected, they were interpolated to a length of 100 frames corresponding to a full gait cycle, normalized from 0 to 100%. An average curve was then computed for both the OMC and IMC data, along with the standard deviation for each frame of the steps. Subsequently, the average gait cycle was computed for both the OMC and IMC datasets, along with the standard deviation at each frame. The number of steps used to generate the average curve was identical for both systems and is reported in Table 2. Statistical analysis was then conducted on the resulting average curves.

The mean absolute error (MAE) and root mean square error (RMSE) were calculated to assess the overall difference in amplitude between the OMC and IMC curves. To assess the similarity in curve shape regardless of differences in amplitude or offset, the signals were first z-score normalized before computing the Pearson correlation coefficient (r). Additionally, 95% confidence intervals (CI), including lower and upper CI bounds, were reported to estimate the reliability and variability of the comparisons.

## 3. Results

The heterogeneity of the dataset, due to the varied walking capacities across groups, was valuable for assessing the robustness of the REEV SENSE IMUs. A total of 517 steps were recorded across 20 participants (mean: 25.8 steps per participant; see Table 2). The “None” group recorded 206 steps (mean: 20.6), the “Cane” group 272 steps (mean: 34.0), and the “Walker” group 39 steps (mean: 19.5).

**Figure 3:**
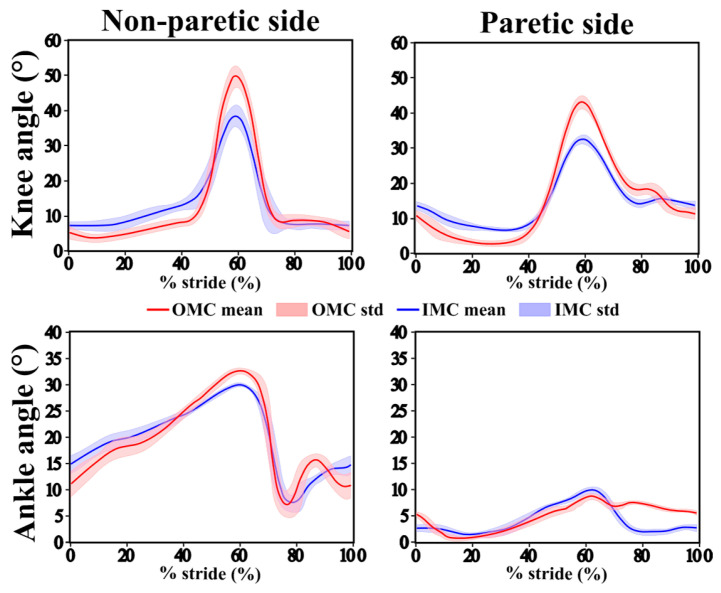
Mean joint angle trajectories (solid line) and standard deviations (shaded area) for the OMC system (red) and IMC system (blue) over the gait cycle for the knee (**top** row) and ankle (**bottom** row) on the non-paretic side (**left**) and paretic side (**right**) for one participant.

Figure 3 shows the representative average curves of the knee and ankle flexion/extension angles over the gait cycle, comparing the IMC and OMC data. While global patterns were similar, differences in amplitude were observed, particularly at peak knee flexion during the swing phase, where the IMUs tended to underestimate the joint angle. The ankle curves showed smaller discrepancies between the IMC and OMC systems. While they do not fully reflect the variability observed across the entire cohort, particularly given the heterogeneity in gait patterns due to varying pathologies, the participant shown in Figure 3 was selected because their data provided a clear and visually interpretable example of typical waveform patterns, making it suitable for illustrative purposes.

The average knee range of motion (ROM) was consistently higher when measured with OMC than with IMC across all subgroups (Table 3). On both sides, the differences were higher in the Cane group (21.49% for the non-paretic side and 34.12% for the paretic side). The lowest difference was found in the Walker group on the paretic side (3.74%).

For the ankle, ROM values were also lower with IMC, but the discrepancy was more pronounced in the Cane and Walker groups. On the non-paretic side, the lowest difference was found in the None group (difference, 2.07%). On the paretic side, the OMC and IMC measurements differed more than those on the non-paretic side. Reduced ROM may reflect clinically meaningful deviations that could influence clinical decision-making.

Overall, OMC tended to yield higher ROM values than IMC, especially for the knee joints, and more pronounced differences were observed in participants requiring walking aids. The reduced ROM measured by the IMC suggests that the system may underestimate joint motion, possibly due to soft tissue artifacts, sensor misalignment, or limitations in the orientation estimation algorithm, especially in pathological gait.

In biomechanics, an MAE (Mean absolute error) lower than 5° is considered clinically acceptable [20]. The lowest MAE was observed for the non-paretic knee with the cane (3.35°), and the highest was observed with the walker (4.30°), with an overall average of 3.81°. The paretic knee showed higher errors, ranging from 4.50° (Cane) to 5.31° (Walker), with an average of 4.95° (All).

At the non-paretic ankle, MAE varied more widely depending on the assistive device, from 3.41° (None) to 7.05° (Walker). The paretic ankle showed generally higher MAE values, ranging from 2.73° (cane) to 8.43° (walker), with an overall average of 3.85°.

Overall, the use of a walker was associated with higher errors, while the cane tended to improve estimation accuracy, particularly for the knee, probably because it improves stability and reduces movement variability, making joint motion more consistent and easier to predict.

The root mean square error (RMSE), representing the average error between the IMC and OMC measurements, remained below 5° for most joints and walking conditions (Table 4). For the knee on the non-paretic side, the RMSE values ranged from 4.23° ± 1.49° in the Cane group to 6.25° ± 4.19° in the Walker group. On the paretic side, the knee showed a minimum RMSE of 4.41° ± 0.41° in the Walker group.

Some RMSE values exceeded the 5° threshold defined in the introduction (12.65° for the walker group), indicating that the accuracy may be limited for certain assistive devices. However, across all conditions, the average RMSE values remained below 5° for both ankles.

The 95% CI bounds of the curve differences were mostly centered on zero, indicating a lack of systematic bias (Table 4). However, in the Walker group, these intervals were not centered around zero ([−12.12; −9.51] for the ankle on the paretic side), indicating reduced consistency between the two systems for this subgroup. The poor agreement in the walker group may have been due to device interference, inconsistent use, or altered gait patterns.

Correlation strength was interpreted using common thresholds in medical research: values between 0.5 and 0.7 indicate moderate to good correlation, 0.7 to 0.9 reflect strong correlation, and values above 0.9 are considered very strong or excellent. These thresholds are widely used to assess model agreement and measurement validity in biomechanics [21].

The strongest correlations were observed in the non-paretic knee, with average Pearson’s r values exceeding r = 0.9 across the three cohorts, reflecting excellent agreement between IMC and OMC measurements (Table 4).

On the paretic knee, the correlations remained strong, with average r values above r = 0.8. When considering all participants, the average correlation in the non-paretic ankle was r = 0.57, while it was slightly higher on the paretic side, reaching r = 0.77. Lower correlations were observed in the ankle joints. In particular, the Cane cohort showed the lowest average correlation on the non-paretic ankle (r = 0.29), meaning that the relationship between the OMC and IMC systems is present but not strong. This suggests that other factors likely influence the outcome; therefore, conclusions should be cautious.

The None group exhibited the most reliable results on both sides for both joints, while the least reliable results were found for the Walker group. The cane group showed good agreement on all sides and joints, except for the ankle on the non-paretic side.

## 4. Discussion

This study aimed to validate the REEV SENSE IMUs against the gold-standard OMC method for capturing lower limb joint angles during pathological gait.

Healthy adults typically show ROM of 60–70° for the knee and 25–30° for the ankle [22,23]. In our dataset, the ROM of both joints on both sides was lower than that of healthy adults, which confirmed the impact of hemiparesis induced by strokes on joint mobility. The average OMC knee ROM on the non-paretic side was 51.05° ± 8.01° for participants without assistance and 45.86° ± 14.6° overall. On the paretic side, the average knee ROM was higher: 53.69° ± 15.23°. Ankle ROM showed a pronounced reduction: the non-paretic ankle reached only 21.88° ± 8.74° on average, while the paretic ankle reached 17.82° ± 8.26°.

The observed correlation patterns showed that the IMC system performed differently across the joints and conditions. The non-paretic knee yielded the strongest correlations (0.93 ± 0.26 for All), suggesting that the IMC reliably captures sagittal movements in less impaired limbs, especially during larger, well-defined motions, such as knee flexion. This also suggests that IMC reliability is higher when motor control is better. The paretic knee also showed good agreement (r = 0.84 ± 0.28 for All), although it was slightly lower. In contrast, correlations were consistently weaker at the ankle, particularly on the non-paretic side in the Cane group (r = 0.29). This drop likely stems from the smaller range of motion and increased variability at the ankle joint [24], compounded by external supports, such as walkers, that modify the natural gait. Notably, the paretic ankle displayed slightly higher correlations (r = 0.77 ± 0.40 for All) than the non-paretic ankle (r = 0.57 ± 0.46 for All), possibly due to more constrained movement patterns in severely affected limbs, which may be easier to model. Further analysis could explore why the cane group exhibited the lowest ankle r, potentially indicating distinct biomechanical adaptations or compensatory strategies in this subgroup.

These trends highlight a key limitation: while IMUs offer strong potential for assessing major joint movements like knee flexion, they are less robust in capturing fine distal motions, especially when assistive devices or pathological variability are involved. This highlights the importance of cautiously interpreting distal joint measurements.

Notably, the largest discrepancies between the IMC and OMC systems tended to occur near the peak joint angles. This pattern has been observed in earlier studies and may be due to difficulties in the sensors when movements are very fast or change direction quickly, which can challenge the way they combine data to estimate motion [25]. Peaks in joint motion are clinically critical for assessing the full range of motion and detecting subtle gait abnormalities that may indicate early functional deficits or guide rehabilitation strategies. Inaccuracies at these moments may reduce the sensitivity of IMU-based assessments, particularly in clinical contexts where precise detection of impairments is necessary. However, the REEV SENSE IMUs used in this study demonstrated low absolute errors (MAE < 5°) and CIs centered around zero, supporting their clinical viability for accurately capturing joint motion in real-world settings.

The walker group consisted of only two participants, which limited the robustness of the statistical conclusions. Nonetheless, this group consistently exhibited the lowest agreement between the IMC and OMC systems across all evaluated metrics. While this may reflect genuine challenges in measuring pathological gait, the small sample size likely contributed to increased variability and may have exaggerated observed discrepancies. This might also indicate that the walker group may fall outside the typical use case for REEV SENSE IMUs due to their atypical gait patterns. However, these results should be interpreted with caution.

REEV SENSE IMUs have shown strong potential for clinical use, offering a compact and easy-to-deploy alternative to traditional motion-capture systems. This makes them well-suited for use in outpatient settings or during routine clinical assessments. In particular, they enable the objective monitoring of joint kinematics during gait, which is essential for evaluating motor recovery in post-stroke patients. This facilitates more frequent assessments and the extraction of longer-term trends without the need for specialized laboratory infrastructure, thereby supporting personalized rehabilitation strategies. This also could enable remote, continuous monitoring of motor recovery over time, supporting longitudinal tracking in a real-world clinical environment.

The first hypothesis of this study was that IMC-based motion-capture systems could provide accurate estimations of lower limb joint angles compared to OMC. Our results support this hypothesis for most joints and conditions, with MAE values below the 5° threshold commonly cited in the literature for acceptable system validation [26,27]. This indicates that, in post-stroke participants, REEV SENSE IMUs can track joint kinematics, making them suitable for clinical use and potentially outside laboratory settings.

Our second hypothesis, that the use of assistive devices would influence the accuracy of IMU-based estimations, was confirmed. Participants using walkers, in particular, exhibited higher MAE values and lower Pearson’s r correlation values, especially at the ankle joint. Assistive devices may interfere by causing upper-body sway, which affects sensor stability, or by creating contact points that alter natural movement patterns, both of which can reduce IMU measurement accuracy. These results highlight the limitations of IMUs with severely altered gait patterns, like in individuals using walkers, and emphasize the need for cautious interpretation since this group was small within the pathological cohort.

One important limitation of this study was the sample size, especially in the walker group (N = 2), which limited the statistical power and precluded strong conclusions regarding the results for this population. The presence of a walker introduced motion artifacts and altered typical gait patterns, potentially reducing the accuracy of IMU-based estimates. Future studies with larger and more balanced groups are required to strengthen the observed trends.

Another concern is IMU calibration and placement, which remain critical sources of variability. Even small misalignments or shifts can affect joint angle estimations, especially for multi-plane rotations. This issue is common across IMC systems, despite standardization efforts. Future work could explore calibration-insensitive algorithms to reduce such errors. Additionally, validation across varied gait conditions like turning and stair climbing should be the next step to ensure robustness in diverse real-life movements.

Additionally, the comparison with OMC, which is considered the gold standard, has its own limitations. Marker placement, soft tissue artifacts, and filtering choices in the OMC data treatment pipeline could also introduce errors. Thus, these differences may stem from combined system limitations rather than errors that are specific to the IMUs.

Moreover, our analysis focused on the sagittal plane in order to capture the dominant forward motion patterns, but may have overlooked discrepancies in frontal and transverse movements. Given that out-of-plane errors might occur with IMUs due to drift and magnetic disturbances, future work should extend to the validation of multiple planes.

Finally, we evaluated the IMU performance in a controlled indoor environment. Magnetic disturbances near metal objects tend to have the greatest impact on IMU accuracy, followed by uneven surfaces and changes in walking speed. However, as the primary use case targets clinical settings with trained professionals, some of these limitations can be mitigated through operator training, standardized IMU placement, and controlled testing environments, making the system well-suited for supervised physical therapy assessments.

These findings suggest that REEV SENSE IMUs demonstrated strong agreement with OMC and are suitable for monitoring lower limb kinematics in post-stroke individuals, providing a practical and portable solution compared to traditional OMC systems. Their ease of use, portability, and strong agreement with gold-standard systems make them well-suited for routine clinical assessments and gait evaluations. These REEV SENSE IMUs can support more frequent, flexible, and patient-centered evaluations in clinical environments.

## 5. Conclusions

This study evaluated the accuracy of the REEV SENSE IMUs in estimating lower limb joint kinematics during gait using an optical motion capture system as a reference. The IMU system showed strong agreement with the optical system, with Pearson’s r often reaching or exceeding 0.9 for the knee, MAE remaining below 5° for the knee and ankle, and 95% confidence intervals centered around zero and within ±2° across all joints and participant groups. These results support the validity of the REEV SENSE IMUs for measuring joint angles during walking, including in post-stroke individuals. However, reduced accuracy was noted in participants who used walkers. Overall, this study highlights the potential of IMU systems as reliable tools for wearable gait analysis while emphasizing the need for caution in more complex or atypical movement scenarios. These findings support the potential use of REEV SENSE IMUs in clinical settings, particularly for rehabilitation planning.

## Figures and Tables

**Table 1:** Participant demographics, with mean ± standard deviation.

Assist.	N	Height (m)	Age (y. o.)	Weight (kg)
None	10	1.74 ± 0.09	55.20 ± 9.34	76.43 ± 9.704
Cane	8	1.67 ± 0.10	54.80 ± 13.19	74.78 ± 18.86
Walker	2	1.80 ± 0.22	54.78 ± 9.19	96.16 ± 24.70
All	20	1.71 ± 0.11	55.44 ± 11.61	75.75 ± 16.73

**Table 2:** Step counts per participant categorized by assistive device use. The sum and mean number of steps for each classification and in total are also reported.

	None									
Participant number	P.1	P.2	P.3	P.4	P.5	P.6	P.7	P.8	P.9	P.10
Number of steps (steps)	23	10	15	12	10	28	17	14	59	18
	Sum = 206, Mean = 20.6									
	Cane								Walker	
Participant number	P.11	P.12	P.13	P.14	P.15	P.16	P.17	P.18	P.19	P.20
Number of steps (steps)	40	66	13	70	20	32	17	14	25	14
	Sum = 272, Mean = 34.0								Sum = 39.0 Mean = 19.5	
	Total Sum = 517, Total Mean = 25.8									

**Table 3:** Range of motion (ROM) for OMC- and IMC-based measurements of the knee and angle for paretic and non-paretic sides. Mean and (standard deviation) are expressed in degrees for the different cohorts.

		None (N = 10)	Cane (N = 8)	Walker (N = 2)	All (N = 20)
Non-paretic Knee	OMC ROM (°)	51.05 ± 8.01	38.33 ± 12.83	61.33 ± 18.8	45.86 ± 14.60
	IMC ROM (°)	37.51 ± 5.88	26.26 ± 9.70	47.18 ±19.4	33.00 ± 12.54
	Difference (%)	26.52	31.49	23.07	28.04
Paretic Knee	OMC ROM (°)	60.69 ± 12.87	50.85 ± 9.75	45.43 ± 32.22	53.69 ± 15.23
	IMC ROM (°)	46.14 ± 9.60	33.50 ± 8.54	43.73 ± 20.76	40.49 ± 11.54
	Difference (%)	23.97	34.12	3.74	24.59
Non-paretic Ankle	OMC ROM (°)	28.06 ± 4.00	18.86 ± 9.88	16.90 ± 0.90	21.88 ± 8.74
	IMC ROM (°)	27.48 ± 5.71	18.36 ± 7.52	19.86 ± 1.88	21.76 ± 7.61
	Difference (%)	2.07	2.65	17.51	0.55
Paretic Ankle	OMC ROM (°)	20.52 ± 5.42	15.79 ± 8.66	15.23 ± 10.77	17.82 ± 8.26
	IMC ROM (°)	24.51 ± 9.40	12.45 ± 5.54	20.12 ± 14.23	18.21 ± 10.01
	Difference (%)	19.44	21.15	32.11	2.19

**Table 4:** Statistical comparison between IMC- and OMC-based joint angles. Mean ± standard deviation of MAE (mean absolute error), RMSE (Root Mean Square Error), 95% CI (Confidence Interval) bounds (CI up and CI low), and Pearson’s correlation parameter (r) are reported for ankle and knee flexion on the paretic and non-paretic sides for the three cohorts. The red values indicate poor agreement.

		Non-Paretic Side				Paretic Side			
		None (N = 10)	Cane (N = 8)	Walker (N = 2)	All (N = 20)	None (N = 10)	Cane (N = 8)	Walker (N = 2)	All (N = 20)
Knee	MAE (°)	4.31 ± 1.24	3.35 ± 0.67	4.30 ± 1.02	3.81 ± 1.01	5.29 ± 1.91	4.50 ± 1.21	3.71 ± 1.33	4.95 ± 1.79
	RMSE (°)	4.78 ± 1.07	4.23 ± 1.49	6.25 ± 4.19	4.64 ± 1.70	5.87 ± 2.24	5.30 ± 1.50	4.41 ± 0.41	5.45 ± 1.79
	CI up (°)	0.86 ± 1.17	0.88 ± 1.51	−3.01 ± 5.69	0.58 ± 2.16	1.48 ± 3.87	2.39 ± 3.09	−1.86 ± 5.72	1.54 ± 3.80
	CI low (°)	−1.15 ± 0.84	−0.94 ± 1.14	−5.24 ± 6.73	−1.36 ± 2.17	−2.15 ± 3.07	−0.33 ± 2.36	−4.53 ± 5.51	−1.56 ± 3.21
	r	0.95 ± 0.03	0.90 ± 0.28	0.98 ± 0.56	0.93 ± 0.26	0.85 ± 0.30	0.82 ± 0.29	0.86 ± 0.2	0.84 ± 0.28
Ankle	MAE (°)	3.41 ± 1.31	2.11 ± 0.86	7.05 ± 2.35	3.22 ± 1.61	4.28 ± 1.50	2.73 ± 1.60	8.43 ± 5.32	3.85 ± 2.68
	RMSE (°)	4.74 ± 3.01	5.15 ± 3.67	5.06 ± 2.92	4.99 ± 3.45	3.78 ± 1.93	2.92 ± 1.59	12.65 ± 7.52	4.16 ± 3.17
	CI up (°)	2.00 ± 2.00	1.52 ± 3.97	−0.81 ± 0.47	1.70 ± 3.01	1.82 ± 3.03	0.60 ± 0.55	−9.51 ± 5.74	0.37 ± 3.47
	CI low (°)	−0.20 ± 1.15	0.45 ± 3.68	−2.70 ± 1.56	−0.11 ± 2.58	−0.29 ± 1.76	−0.64 ± 0.54	−12.12 ± 7.25	−1.43 ± 3.30
	r	0.72 ± 0.36	0.29 ± 0.40	0.51 ± 0.29	0.57 ± 0.46	0.78 ± 0.35	0.78 ± 0.45	0.61 ± 0.35	0.77 ± 0.40

## Data Availability

The datasets presented in this article are not readily available because the data are part of a clinical study named REEV SENSE for gait analysis in post-stroke gait impairment (SENS-AG) (NCT Num-ber: NCT06234878). Requests to access the datasets should be directed to the corresponding author.

## Abbreviations

The following abbreviations are used in this manuscript:

OMC	Optical motion capture
IMC	Inertial motion capture
MAE	Mean absolute error
RMSE	Root mean square error
IMU	Inertial Measurement Unit
HGA	Human gait analysis
RDSF	Relative distance between sacral and foot markers
ROM	Range of motion
